# Masked face identification is improved by diagnostic feature training

**DOI:** 10.1186/s41235-022-00381-x

**Published:** 2022-04-05

**Authors:** Daniel J. Carragher, Alice Towler, Viktoria R. Mileva, David White, Peter J. B. Hancock

**Affiliations:** 1 Psychology, Faculty of Natural Sciences, University of Stirling, Stirling, Scotland, UK; 2 School of Psychology, University of New South Wales, Sydney, NSW Australia; 3 Present Address: School of Psychology, Faculty of Health and Medical Sciences, University of Adelaide, Adelaide, SA 5000 Australia

**Keywords:** Facial image comparison, Face recognition, Face matching, Masks, COVID-19, Knowledge elicitation

## Abstract

To slow the spread of COVID-19, many people now wear face masks in public. Face masks impair our ability to identify faces, which can cause problems for professional staff who identify offenders or members of the public. Here, we investigate whether performance on a masked face matching task can be improved by training participants to compare diagnostic facial features (the ears and facial marks)—a validated training method that improves matching performance for unmasked faces. We show this brief diagnostic feature training, which takes less than two minutes to complete, improves matching performance for masked faces by approximately 5%. A control training course, which was unrelated to face identification, had no effect on matching performance. Our findings demonstrate that comparing the ears and facial marks is an effective means of improving face matching performance for masked faces. These findings have implications for professions that regularly perform face identification.

**Supplementary Information:**

The online version contains supplementary material available at 10.1186/s41235-022-00381-x.

## Significance statement

The ongoing COVID-19 pandemic is signified by the face masks many people now wear in public. This mask wearing can pose problems for professional staff who need to identify people from their facial appearance, such as shop assistants who might compare a shopper to their photo-ID, or police officers who identify suspects from CCTV footage. This task is surprisingly difficult at the best of times, as the average person makes 20–35% errors when trying to identify unmasked unfamiliar faces. Unsurprisingly, errors increase when one of the faces is shown wearing a face mask, which typically covers the nose, mouth, and chin. Here, we build on previous research showing accuracy benefits after instructing participants to focus on the ears and facial marks of the two faces when performing unfamiliar face matching. Because these features often remain visible while wearing a mask, we predicted that this diagnostic feature training would also improve face matching performance when one face in the pair is shown wearing a mask. Our results supported this prediction. We found that a two-minute diagnostic feature training course improved people’s masked face matching performance by approximately 5%. Professional staff who are required to identify masked faces would benefit from completing diagnostic feature training.

## Introduction

The COVID-19 pandemic has led to a sudden and remarkable increase in the number of people wearing face masks^1^ in public, an otherwise uncommon choice in many countries (Morning Consult, 2020; YouGov, 2020). Public tracking polls from March 2020 show that even at the outset of the pandemic, very few respondents from Australia (10%), the UK (1%) and the USA (7%) reported wearing a face mask in public, compared to 62% of respondents from Japan (YouGov, 2020), where public mask wearing was already common (Horii, 2014). Over the course of the pandemic, the same poll has reported peak mask wearing of 70% in Australia (July 2021), 77% in the UK (February 2021), 83% in the USA (November 2020), and 86% in Japan (May 2020). Although these increases were almost certainly due to the mandated wearing of face masks in public spaces (#Masks4All, 2021; Centers for Disease Control & Prevention, 2020), there are early indications that many individuals intend to continue wearing face masks in public, even when they are no longer legally required to do so (Office for National Statistics, 2021).

The increased prevalence of mask wearing is problematic in applied situations where faces are used for identity verification, for example, in law enforcement and security settings (Babwin & Dazio, 2020). Although the vast majority of people who wear face masks into stores do so to follow the recommendations of public health agencies (Centers for Disease Control & Prevention, 2020), there have also been reports of individuals exploiting this expectation by committing crimes while wearing face masks (Southall & Van Syckle, 2020). Recent research has shown that these masks disrupt normal face processing, making it harder to identify both familiar and unfamiliar people (Carragher & Hancock, 2020; Freud et al., 2020; Noyes et al., 2021). While it is possible that we may adapt to this change over time, preliminary evidence suggests that natural exposure to masked faces throughout the course of the pandemic has not yet improved our ability to accurately identify masked faces (Freud et al., 2021). Since the number of people wearing face masks in public will likely remain elevated for the duration of the pandemic, and possibly beyond (Horii, 2014; Office for National Statistics, 2021), finding ways to improve identification accuracy for masked faces is of critical importance for national security and the criminal justice system.

Even unmasked, correctly identifying unfamiliar faces is surprisingly difficult (Bruce et al., 1999; Kemp et al., 1997). When asked to decide whether two simultaneously presented faces show the same person or two different people, the average observer makes errors on approximately 20% of trials under the most ideal circumstances, such as when the two photographs are taken on the same day in controlled studio settings (Burton et al., 2010). However, even slight differences in lighting (Hill & Bruce, 1996), viewpoint (Estudillo & Bindemann, 2014), or the distance between the camera and the model (Noyes & Jenkins, 2017), further impair unfamiliar face matching performance (Fysh & Bindemann, 2017b), as does the amount of time that has passed between capturing the two photographs (Megreya et al., 2013), or whether the images are shown in colour or greyscale (Bobak et al., 2019). As such, error rates in tests that are more representative of applied settings can often exceed 30% (Carragher & Hancock, 2020; Dowsett & Burton, 2015; Fysh & Bindemann, 2018). Similarly high error-rates are observed among many professional groups (see White et al., 2021 for a meta-analysis), despite years of experience (White et al., 2014b) and standard industry training (Towler et al., 2014, 2019).

Perhaps unsurprisingly, face masks cause further impairment to human performance on tasks of face recognition (Freud et al., 2020; Mansour et al., 2020) and matching (Carragher & Hancock, 2020; Dhamecha et al., 2014; Estudillo et al., 2021; Noyes et al., 2021). Compared to unmasked faces, Carragher and Hancock (2020) found that matching performance for masked faces declined by 34–52%, regardless of whether one or both faces in the pair wore masks, or whether the faces were familiar or unfamiliar to the observer. Noyes et al. (2021) extended this line of research to show that while “super-recognizers”—people with extraordinary face recognition abilities (Russell et al., 2009)—still outperformed control participants on a masked face matching task, the performance of both groups was equally impaired by masks. Taken together, these findings suggest that face masks cause a relatively consistent impairment to matching performance, regardless of the familiarity of the faces (Carragher & Hancock, 2020) or the abilities of the observer (Noyes et al., 2021).

To improve masked face identification, we must first consider *why* face masks impair performance. While this question remains an area of active research, early evidence points to the contributions of two related factors. First, masks might impair accuracy simply because they reduce the amount of identity information available to observers (Davies et al., 1977; McKelvie, 1976). With less of the face visible, there are fewer opportunities for the observer to detect the similarities or differences in appearance that can be useful for identification. Second, masks may reduce accuracy because they disrupt normal holistic face processing (Freud et al., 2021; Stajduhar et al., 2021), whereby faces are perceived as unified wholes rather than a collection of facial features (Maurer et al., 2002; Tanaka & Farah, 1993). Considering these two factors, training interventions that do not rely on whole face processing, but rather, encourage observers to extract maximal identity information from the available visual information, might be particularly well suited to the challenge of improving masked face identification performance.

Diagnostic feature training, a method recently developed by Towler et al. (2021b), is a promising candidate for improving masked face identification performance. Towler et al.’s training teaches novices to focus on the facial features that are most diagnostic of identity for professional facial examiners—specialist professionals who consistently outperform novices on face matching tasks by using a feature-based comparison strategy (Towler et al., 2017; White et al., 2021). Towler et al. (2017) asked professional facial examiners to rate the similarity of 11 facial features on face pairs, and then calculated the extent to which those similarity ratings discriminated between identity match and mismatch pairs. Facial examiners’ similarity ratings of ears and facial marks (e.g. scars, moles, freckles) best predicted the correct answer to each trial, indicating these features are most diagnostic of identity (Towler et al., 2017). Importantly, novices undervalued the importance of these features. Using the expert knowledge elicited from that study, Towler et al. (2021b) developed a “diagnostic feature training course” to teach novices to compare these high-value features—the ears and facial marks—when making their matching decisions. Completing this training improved novices’ accuracy by 6%, which accounts for almost half the accuracy advantage of professional facial examiners (Towler et al., 2021b).

The success of diagnostic feature training stands in clear contrast to many previous attempts to improve unfamiliar face matching performance, which have generally been unsuccessful (for review, see Towler et al., 2021a). For example, professional training programs, which can take hours or days to complete, are largely ineffective (Towler et al., 2014, 2019). The two previously successful approaches, completing the task in a collaborative pair (Dowsett & Burton, 2015), and giving observers feedback about the accuracy of their decisions in real time (White et al., 2014a; however, see Alenezi & Bindemann, 2013), both led to a minor improvement in performance that was limited only to the lowest performing individuals. Crucially, neither approach gives the observers explicit directions about how to improve their performance; rather, both rely on the novice observers creating unvetted strategies to decipher why each pair is or is not an identity match (Dowsett & Burton, 2015; White et al., 2014a). For this reason, neither approach is well suited to the challenge of matching masked faces. In contrast, diagnostic feature training leads to generalised improvement in unfamiliar face matching performance (Towler et al., 2021b), and also neatly fits our criteria for a candidate training intervention to improve masked face matching performance because it does not rely on whole face processing, but rather, directs observers to focus on important features that often remain visible on masked faces.

The aim of the current study was to determine whether diagnostic feature training could also improve face matching performance for unfamiliar masked faces. All participants in this pre-registered experiment completed a face matching task wherein one image in each pair was shown with a mask superimposed over the lower half of the face. Midway through the task, participants were randomly assigned to complete one of two training courses created by Towler et al. (2021b): diagnostic feature training (ears and facial marks), or control training (irrelevant conflict resolution strategies). Since face masks do not obscure the ears or any facial marks in the top half of the face, we expected that directing observers’ attention to these overlooked features through diagnostic feature training would improve matching performance.

## Method

### Sample size

Towler et al. (2021b) reported a significant interaction between *test* (2: pre-training, post-training) and *training condition* (3: diagnostic feature, non-diagnostic feature, control) on the measure of area under the curve (AUC; Green & Swets, 1966) with an effect size of $\eta_{p}^{2}$ = 0.15. An a priori power analysis (G*Power; Faul et al., 2007) with an arbitrarily lowered expected effect size^2^ of $\eta_{p}^{2}$ = 0.10 showed that a total sample of 74 participants was required to achieve 80% power to detect an effect in a mixed-measures ANOVA with *test* (within-participants; pre-training, post-training) and *training condition* (between-participants; diagnostic feature, control) as factors at a conventional alpha of *α* = 0.05. To account for participant exclusions, we aimed to recruit 50 participants to each training condition, so that data from approximately 40 participants would be available in each condition for the final analysis.

### Participants

We recruited 100 participants that completed the experiment from the online research platform *Prolific* (https://www.prolific.co/). All participants were aged 18 years or older and reported living in the UK. To maintain data integrity, we applied several pre-registered exclusion criteria to the collected data prior to analysis. Participants who attempted the experiment more than once^3^ (*n* = 2), took less than 10 min (*n* = 4) to complete the experiment, or failed an attention check trial (*n* = 4) were excluded from all analyses.^4^

The final sample consisted of 90 participants: 46 in the diagnostic feature training condition (32 female, 13 male, 1 other; *M*_age_ = 36.0, *SD* = 13.9, range = 19–66), and 44 in the control training condition (26 female, 17 male, 1 response withheld; *M*_age_ = 34.9, *SD* = 12.3, range = 19–64). This research was approved by the General University Ethics Panel at the University of Stirling. All participants gave their informed consent before starting the experiment, were debriefed on completion, and received £3.00 for their time.

### Expertise in facial comparison test

Participants completed the expertise in facial comparison test (EFCT; White et al., 2015), which consists of images from *The Good, The Bad, and The Ugly* challenge stimulus set (Phillips et al., 2011). Subjects in this image set were photographed multiple times on different days in unconstrained naturalistic settings, ensuring superficial characteristics such as clothing and hairstyle do not cue identity. The face pairs selected for the EFCT were those that had high error rates among computer algorithms and human observers (O'Toole et al., 2012; White et al., 2015). The EFCT contains both male and female face pairs and consists of 168 trials in total.

Like Towler et al. (2021b), we divided the EFCT into two sets of 84 trials known to be of equal difficulty (White et al., 2015). Each set (A, B) had 42 match pairs and 42 mismatch pairs. In the current study, the presentation order (pre-training, post-training) of Set A and Set B was counterbalanced between participants. Within each set, trial order was randomised. The faces were rotated to align the eyes in the centre of the image using custom written code. The stimuli were presented in colour, and each face image was 252 × 357 px in size (approximately 8 × 11.5 cm on a 23″ 1920 × 1080 px monitor).

### Face masks

**Fig. 1:**
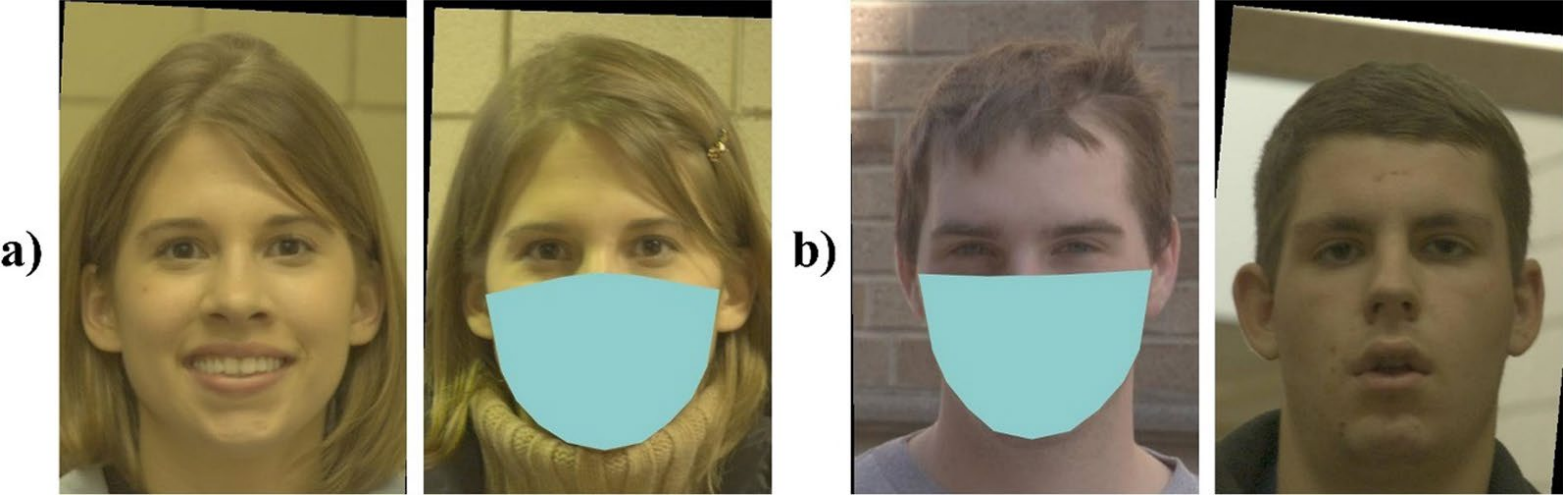
Examples of **a** match and **b** mismatch trials from the EFCT. Participants responded to the question “*Is the same person shown in both photographs?*” using 6 possible responses: “*Definitely Not*”, “*Probably Not*”, “*Guess Not*”, “*Guess Yes*”, “*Probably Yes*”, and “*Definitely Yes*”

We modified the EFCT, such that one face in each image pair always appeared to wear a face mask (see Fig. 1). The masks were plain colour patches that were superimposed over the faces automatically using custom written code. Like real face masks, they were designed to cover the nose, mouth, chin, and jawline of the face. The face in each pair that was masked was selected at random. Across trials, faces on the left and right side of the pairs were masked equally often.

### Attention check

We embedded two attention check trials within the EFCT so that we could screen the data for inattentive or automated participants. These pairs consisted of famous faces that were obvious identity mismatches which, regardless of familiarity, could be distinguished by race (Pair 1: former President Barack Obama & former President Donald Trump) or gender (Pair 2: Queen Elizabeth II & Prime Minister Boris Johnson). These famous faces were presented unmasked. Data from participants who failed to give a response of “*Definitely Not*” to both pairs were discarded from all analyses.

### Training courses

The two training courses were those created by Towler et al. (2021b), where further methodological detail can be found. Briefly, the diagnostic feature training course consisted of 14 slides that instructed participants to compare the ears and any facial marks when making their matching decisions. This training course included labelled images showing the different anatomical features of the ear (e.g. lobe, helix) and different types of facial marks (e.g. moles, freckles), along with example face pairs to illustrate how similarities in these features could be used to infer an identity match. All faces shown in the training course were unmasked. Participants in the control condition completed a 14-slide training course about conflict resolution strategies, which was created using information from the Internet. The control training course offered no information that could conceivably improve face matching performance. Both training courses were self-paced.

### Procedure

The experiment was hosted using Qualtrics survey software. Participants were unable to complete the experiment on a mobile device. All participants were told that their task was to determine whether the two faces in each pair showed the same person. The generic face matching instruction given to all participants at the start of the experiment was “*compare the appearance of the two faces to make your final identity decision*”.

On each trial, two faces were presented on screen simultaneously. Participants made their response to the question “*Is the same person shown in both photographs?*” using a 6-Alternative Forced Choice scale (6AFC: “*Definitely Not*”, “*Probably Not*”, “*Guess Not*”, “*Guess Yes*”, “*Probably Yes*”, and “*Definitely Yes*”). The two faces remained onscreen until a response was made, and there was no time limit on responses. After completing the first half of the EFCT, participants could take a short break before completing their randomly assigned training course (diagnostic feature or control). All participants then completed the second half of the EFCT. The experiment took an average of 22 min (*SD* = 8.2) to complete.

### Analysis

The 6AFC responses were used to create a receiver operating characteristic (ROC) curve for each participant (Green & Swets, 1966; Macmillan & Creelman, 2004). The shape of the ROC is given by plotting the proportion of hits (correctly responding “yes” on a match trial) against false alarms (incorrectly responding “yes” on a mismatch trial) cumulatively at each level of confidence (Definitely, Probably, Guess) for each binary identity decision (No, Yes). Calculated from the ROC, the area under the curve (AUC) offers a measure of sensitivity, expressed as a single value, which describes how well participants can distinguish identity match pairs from mismatch pairs across different response thresholds. An AUC of 1.0 indicates perfect performance, whereas an AUC of 0.5 signals chance performance. As per our pre-registration, AUC is our primary measure of performance.

We also report the signal detection measures of d′ (“dee-prime”) and criterion (Macmillan & Creelman, 2004). Like AUC, d′ is a measure of sensitivity that describes how well participants can discriminate between match and mismatch trials. But unlike AUC, d′ is calculated from a single response threshold across all trials. Criterion is a measure of response bias that is used to index participants’ tendency to make one response type over another across all trials. As such, criterion is not a measure of ability or performance per se; rather, it offers an insight into response strategy.

Both measures (d′, criterion) were calculated from hits and false alarms (Stanislaw & Todorov, 1999), which were recorded by collapsing across the confidence component of our 6AFC scale, leaving only “yes” and “no” responses to each trial (i.e. “Definitely Yes”, “Probably Yes” and “Guess Yes” were all counted as “yes”). With a necessary correction for extreme performance (Stanislaw & Todorov, 1999), 4.52 is the maximum value of d′ possible in each half of the EFCT. A d′ of 0 indicates chance performance. Criterion ranges from − 2.26 to 2.26 for each half of the EFCT. Negative criterion values indicate a bias to report “yes” (a liberal criterion), while positive values indicate a bias to report “no” (a conservative criterion). Neutral responding is indicated by a criterion value of 0.

For completeness, we also report a full analysis of accuracy as a secondary measure. The purpose of this additional analysis is to facilitate the translation of this research to applied settings by providing a more concrete estimate of effect sizes, while also ensuring that our results are more interpretable within a policy context. Here, we include an analysis of overall accuracy, as well as separate analyses for match and mismatch trials, because performance across the two trial types is only weakly correlated (Megreya & Burton, 2007).

As per our pre-registration, we have supplemented the frequentist *t*-tests in our planned and simple main effects analyses with equivalent Bayesian *t*-tests. Unlike frequentist analyses, Bayesian analyses can provide evidence in favour of the alternative (BF_10_) or null (BF_01_) hypotheses, and their interpretation is unaffected by sample size (Wagenmakers et al., 2018). This approach was reported in Towler et al. (2021b) original diagnostic feature training paper and is employed again here for consistency and to allow comparison. The following classification scheme (JASP Team, 2020) can be used to characterise the strength of our Bayes factors (Goss-Sampson et al., 2020), which are all reported as BF_10_ values. Bayes factors of 1–3, 3–10 and > 10 provide anecdotal, moderate and strong evidence, respectively, in favour of the *alternative* hypothesis. Values between 1.00–0.33, 0.33–0.10 and < 0.10 provide anecdotal, moderate and strong evidence in favour of the *null* hypothesis. All Bayesian analyses use default priors (JASP Team, 2020).

The aims, hypotheses, design, and analyses for this experiment were pre-registered on the open science framework (OSF) prior to data collection [https://osf.io/qw27y]. Planned (primary) and exploratory (secondary) analyses are clearly identified in the results section below. Each analysis of variance (ANOVA) has *test* (pre-, post-) as a within-participants factor and *training condition* (diagnostic feature, control) as a between-participants factor. All analyses were performed in JASP 0.14.0 (JASP Team, 2020). All data analysed in this study are available on the OSF [https://osf.io/9y24q/].

## Results

### Primary analyses

#### Training course duration

The median time taken to complete the diagnostic feature training course was 100.5 secs (1 min 41 secs), while the median time for the control training was 102.5 secs (1 min 43 secs). An independent samples *t*-test confirmed that average completion time did not differ between the diagnostic feature (*M* = 119.7 secs, *SD* = 65.7) or control training courses (*M* = 134.0 secs, *SD* = 173.4), *t*(88) = 0.52, 95% CI[-40.16, 68.81], *p* = 0.603, *d* = 0.11.

#### AUC

**Fig. 2:**
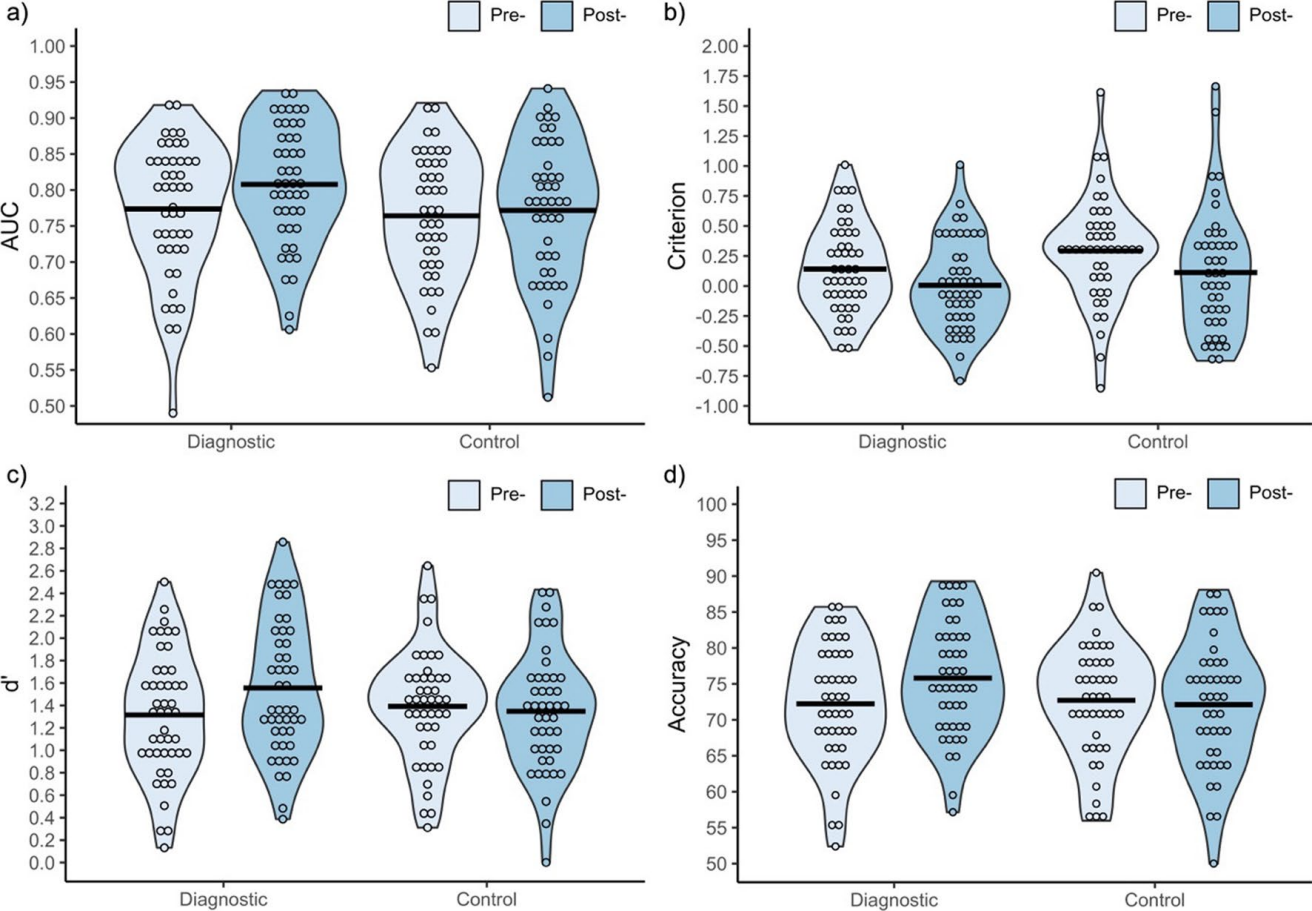
Performance measures pre- and post-training for each training condition. **a** Area under the curve (AUC). **b** Response bias (criterion for declaring a match). **c** Sensitivity (d′), **d** overall accuracy (%). On all figures, unfilled circles represent individual data points (visualised in 1/30 bins by default), while the horizontal black lines represent the mean

A mixed measures ANOVA on AUC showed that the main effect of test was significant, *F*(1, 88) = 7.78, *p* = 0.006, $\eta_{p}^{2}$ = 0.08, due to the higher AUC post-training (*M* = 0.790, *SD* = 0.092) than pre-training (*M* = 0.769, *SD* = 0.091). The main effect of training condition was not significant, *F*(1, 88) = 1.68, *p* = 0.199, $\eta_{p}^{2}$ = 0.02. The interaction between the two factors was non-significant, *F*(1, 88) = 3.19, *p* = 0.078, $\eta_{p}^{2}$ = 0.04 (see Fig. 2a).

Following the approach outlined in our pre-registration, we conducted planned paired samples *t*-tests to compare AUC pre- and post-training for both training conditions. In the absence of a significant interaction, this analysis was designed to address our fundamental research question, which was to discover whether diagnostic feature training improves masked face matching performance. As predicted, there was a significant increase in AUC post-training for the diagnostic feature condition, whereas there was no change for the control condition (see Table 1). From a Bayesian perspective, the increase for the diagnostic condition offers strong support for the hypothesis that diagnostic feature training improves matching performance for unfamiliar masked faces (Lee & Wagenmakers, 2014). Conversely, the data in the control condition offer moderate support in favour of the null hypothesis. Despite this encouraging pattern of results, the non-significant interaction in the ANOVA above prevents us from concluding that diagnostic feature training leads to greater improvement in AUC than the control training course.

**Table 1:** Planned paired samples *t*-tests (AUC) and simple main effects analysis (d′, overall accuracy) comparing mean performance pre-training to post-training for both training conditions

Measure	Training	Pre-training	Post-training	*df*	*t*	95% CI	*p*	*d*	BF_10_
AUC	Diagnostic	.774 (.094)	.808 (.083)	45	3.28	0.01, 0.06	.002*	0.48	15.76
	Control	.764 (.088)	.772 (.099)	43	0.70	− 0.01, 0.03	.487	0.11	0.21
d′	Diagnostic	1.32 (0.56)	1.56 (0.59)	45	3.16	0.09, 0.39	.003*	0.47	11.52
	Control	1.39 (0.51)	1.35 (0.53)	43	− 0.68	− 0.18, 0.09	.501	0.10	0.20
Overall Accuracy	Diagnostic	72.23 (8.21)	75.80 (7.97)	45	3.39	1.45, 5.70	.001*	0.50	20.67
	Control	72.70 (8.04)	72.11 (8.74)	43	− 0.63	− 2.49, 1.30	.530	0.10	0.20

The Bonferroni-corrected alpha for two comparisons is *p* < .025

*Identifies statistically significant comparisons

#### Criterion

A mixed measures ANOVA on criterion revealed a significant main effect of Test, *F*(1, 88) = 14.94, *p* < 0.001, $\eta_{p}^{2}$ = 0.15, with a larger response bias pre-training (*M* = 0.22, *SD* = 0.41) than post-training (*M* = 0.06, *SD* = 0.45). This conservative response bias indicates that at pre-training, participants in both conditions tended to report that pairs showed two different people. The main effect of training condition was non-significant, *F*(1, 88) = 2.54, *p* = 0.115, $\eta_{p}^{2}$ = 0.03, as was the interaction between the two factors, *F*(1, 88) = 0.31, *p* = 0.577, $\eta_{p}^{2}$ = 0.00 (see Fig. 2b). One-sample *t*-tests showed that the response bias of both training conditions differed from neutral pre-training, but not post-training (see Table 2).

**Table 2:** One sample *t*-tests comparing the response bias shown by each training condition to 0, in order to determine whether the response bias differs statistically from neutral responding

Training	Test	Mean (SD)	*df*	*t*	95% CI	*p*	*d*	BF_10_
Diagnostic	Pre-^a^	0.14 (0.37)	45	2.56	0.03, 0.25	.014*	0.38	2.94
	Post-	0.01 (0.37)	45	0.11	− 0.11, 0.12	.910	0.02	0.16
Control	Pre-^a^	0.29 (0.44)	43	4.38	0.16, 0.43	< .001*	0.66	305.37
	Post-	0.11 (0.52)	43	1.44	− 0.05, 0.27	.157	0.22	0.43

^a^A separate independent samples *t*-test confirmed that pre-training criterion did not differ between the two training conditions, *t*(88) = 1.76, 95% CI [− 0.02, 0.32], *p* = .081, *d* = 0.37, BF_10_ = 0.86

*Identifies statistically significant comparisons

### Secondary analyses

#### Sensitivity

A mixed measures ANOVA on d′ showed that the main effects of test, *F*(1, 88) = 3.74, *p* = 0.056, $\eta_{p}^{2}$ = 0.04, and training condition, *F*(1, 88) = 0.40, *p* = 0.528, $\eta_{p}^{2}$ = 0.01, were non-significant (see Fig. 2c). Crucially, the interaction between the two factors was significant, *F*(1, 88) = 7.95, *p* = 0.006, $\eta_{p}^{2}$ = 0.08. Simple main effects analysis revealed there was a significant increase in sensitivity post-training for the diagnostic feature condition, whereas no change occurred for the control condition (see Table 1).

#### Accuracy

##### Overall accuracy

The main effect of test was significant, *F*(1, 88) = 4.41, *p* = 0.039, $\eta_{p}^{2}$ = 0.05, due to higher accuracy post-training (*M* = 74.0%, *SD* = 8.5) than pre-training (*M* = 72.5%, *SD* = 8.1). The main effect of training condition was not significant, *F*(1, 88) = 1.04, *p* = 0.312, $\eta_{p}^{2}$ = 0.01. Crucially, the interaction between test and training conditions was significant, *F*(1, 88) = 8.65, *p* = 0.004, $\eta_{p}^{2}$ = 0.09 (see Fig. 2d). Simple main effects analysis revealed there was a significant increase in overall accuracy post-training for the diagnostic training condition, whereas no change occurred for the control condition (see Table 1).

##### Match trials

**Fig. 3:**
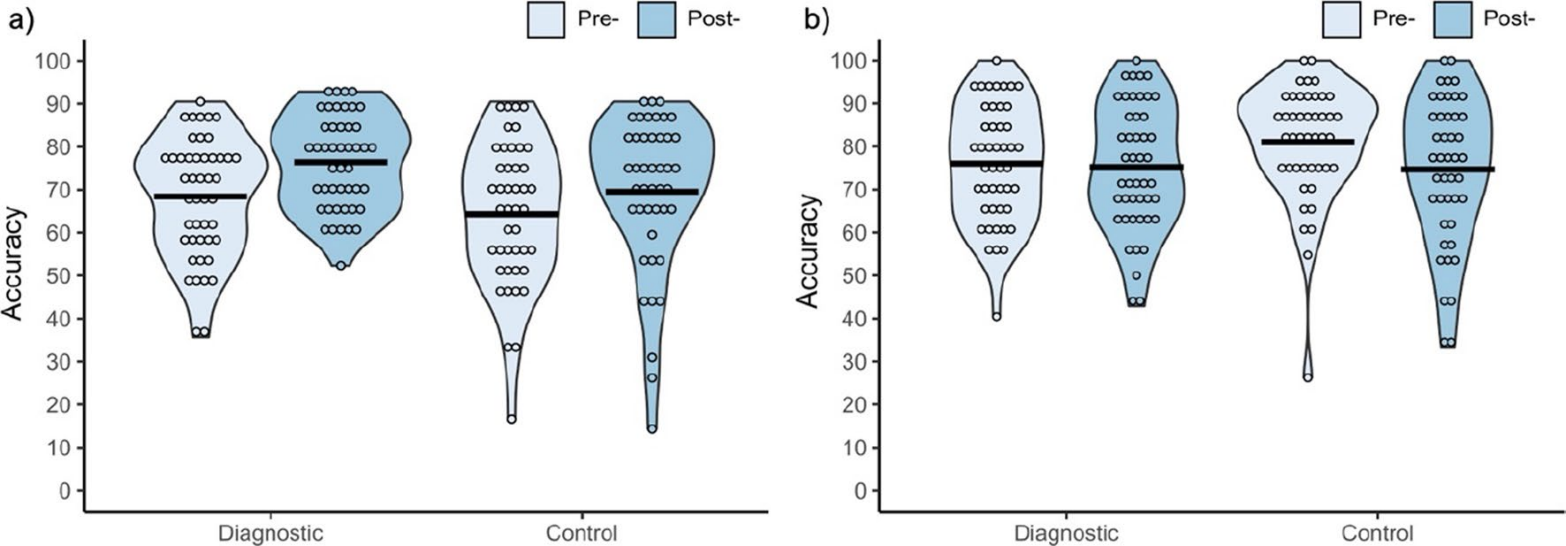
Accuracy (%) on the EFCT for both training conditions on a) match trials b) and mismatch trials

The main effect of test was significant, *F*(1, 88) = 25.12, *p* < 0.001, $\eta_{p}^{2}$ = 0.22, with accuracy higher post-training (*M* = 73.0%, *SD* = 15.0) than pre-training (*M* = 66.4%, *SD* = 15.2). This post-training increase in match trial accuracy is consistent with the liberal response bias shift reported above. The main effect of training condition was non-significant, *F*(1, 88) = 3.66, *p* = 0.059, $\eta_{p}^{2}$ = 0.04, as was the interaction between test and training conditions, *F*(1, 88) = 1.12, *p* = 0.293, $\eta_{p}^{2}$ = 0.01 (see Fig. 3a).

##### Mismatch trials

The main effect of test was significant, *F*(1, 88) = 5.89, *p* = 0.017, $\eta_{p}^{2}$ = 0.06, with higher accuracy pre-training (*M* = 78.5%, *SD* = 13.8) than post-training (*M* = 74.9%, *SD* = 15.8). This post-training decrease in mismatch trial accuracy is consistent with the liberal response bias shift reported above. The main effect of training condition was non-significant, *F*(1, 88) = 0.67, *p* = 0.415, $\eta_{p}^{2}$ = 0.01, as was the interaction between the two factors, *F*(1, 88) = 3.50, *p* = 0.065, $\eta_{p}^{2}$ = 0.04 (see Fig. 3b).

#### Response time

Finally, we investigated whether training influenced median response time (RT). First, an independent samples *t*-test confirmed that median RT did not differ between the two training conditions pre-training, *t*(88) = 1.25, 95% CI [− 0.30, 1.30], *p* = 0.216, *d* = 0.26. A mixed measures ANOVA revealed that the main effect of test was non-significant, *F*(1, 88) = 3.88, *p* = 0.052, $\eta_{p}^{2}$ = 0.04. The main effect of training condition was significant, *F*(1, 88) = 13.79, *p* < 0.001, $\eta_{p}^{2}$ = 0.14, as was the interaction between the two factors, *F*(1, 88) = 37.95, *p* < 0.001, $\eta_{p}^{2}$ = 0.30. Simple main effects analysis revealed that median RT in the diagnostic training condition was slower post-training (*M* = 5.72 secs, *SD* = 2.41) than pre-training (*M* = 4.45 secs, *SD* = 2.06), *F* = 21.03, *p* < 0.001. Conversely, the control condition made faster responses post-training (*M* = 3.30 secs, *SD* = 1.70) than pre-training (*M* = 3.95 secs, *SD* = 1.74), *F* = 23.60, *p* < 0.001.

## Discussion

Participants who completed the diagnostic feature training course (Towler et al., 2021b) improved their sensitivity (d′) and overall accuracy for matching unfamiliar masked faces. Although the interaction term for our primary measure of AUC was non-significant, planned Bayesian *t*-tests showed that the 4.4% increase in AUC for the diagnostic training condition was nearly 16 times more likely to occur if the training course truly improves sensitivity, which is considered strong evidence in favour of an effect (Goss-Sampson et al., 2020). There were no such changes among the control condition, whose data provided moderate evidence in favour of the null hypothesis across these performance measures. Together, these data demonstrate that diagnostic feature training, which instructs observers to compare the ears and any markings on the two faces, is a viable strategy to improve sensitivity (d′), and overall accuracy, when matching unfamiliar masked faces.

Diagnostic feature training led to a 4.9% increase in overall accuracy and a 4.4% increase in AUC. Both increases are similar, albeit slightly smaller, to the 6% gain in AUC previously shown to occur when this training was given to assist matching unmasked faces (Towler et al., 2021b). But a slightly smaller effect for masked faces is entirely consistent with the changed nature of the task. A facial mark only has identification value if the observer can ascertain that it is present or absent on the second image. Thus, any facial marks that lie within the area covered by the mask—even on the unmasked face—lose their identification value, since they either cannot be seen or used for comparison. Nonetheless, our findings suggest that gains in matching performance can be achieved using features outside of the masked area, namely the ears and markings on the upper half of the face.

The conservative response bias shown pre-training by participants in both conditions is consistent with Carragher and Hancock (2020), who also found conservative criterions among participants who completed a matching task with masked faces. Together, these findings suggest that observers are initially reluctant to declare two unfamiliar faces to be an identity match when one is shown wearing a mask (see also Noyes et al., 2021). However, the post-training reduction in conservative bias was unexpected. Since this shift occurred in both conditions, it is likely unrelated to the content of either training course. Instead, this shift is consistent with previous studies of unmasked faces, which show response bias becomes more liberal as time on task increases (Alenezi et al., 2015). With 170 trials in our face matching task, it is likely that this liberal response bias drift also occurred in the current study (Fysh & Bindemann, 2017a). Although this significant response bias shift can affect the interpretation of match and mismatch trial accuracy, measures of sensitivity are independent of response bias because they are calculated from hits and false alarms (Stanislaw & Todorov, 1999). Therefore, the increase in d′ among the diagnostic feature condition cannot be attributed to a shift in response bias, but rather, stems from genuine improvements to their face matching abilities. Future research is needed to investigate whether, and for how long, these performance improvements persist after training.

The improved performance of participants in the diagnostic feature condition post-training coincided with a slowing of their RTs to each trial. But slower RTs are to be expected in this condition, since the participants received instructions to attend to facial features that are often overlooked by novices (Towler et al., 2017), likely requiring additional viewing time (White et al., 2015). While this pattern could also be consistent with a speed accuracy trade-off, the control group’s faster RTs post-training were not associated with a corresponding decrease in accuracy, so we consider this possibility unlikely. The decrease in post-training RT for the control condition is consistent with normal response behaviour in long face matching tasks (Alenezi et al., 2015; also see Additional file 1). Lastly, we note that participants in both conditions took approximately 1 min and 40 s to complete their training courses, whereas Towler et al. (2021b) participants took 5 min and 30 s. Since both studies used the same training courses, recruited participants online, and allowed the training courses to be completed in a self-paced manner, the cause of this discrepancy is unclear. Nonetheless, the performance improvements among the diagnostic feature condition, despite the reduced time spent on training, demonstrate that this particular training course can be completed efficiently in less time than suggested by Towler et al. (2021b).

This diagnostic feature training approach (Towler et al., 2021b) is very similar to the “feature-instruction” approach devised by Megreya and Bindemann (2018), whereby participants received a simple text-based instruction to focus on a particular facial feature when making their matching decision (e.g. “…*please focus on the eyes.*”). Instructing observers to attend to the eyebrows improved performance, whereas attending the eyes had no effect, and attending the ears impaired performance (Megreya & Bindemann, 2018). However, as reported in Additional file 1, we were unable to replicate these results in an online setting using the original (unmasked) version of the EFCT (White et al., 2015), potentially raising questions about the generalisability of the instruction-based approach beyond the original stimulus set (Megreya & Bindemann, 2018). When considered alongside the improvement reported in the main text, this non-replication could indicate that simply directing attention towards any facial feature is not sufficient to reliably improve unfamiliar face matching performance; rather, benefits might only arise when attending to those features that carry diagnostic identity information (Towler et al., 2017). It should also be considered that observers may benefit from the additional detail and pictorial examples that are given in the diagnostic feature training course (Towler et al., 2021b). Further research is needed to examine exactly which components of the diagnostic feature training course are responsible for the improvements in face matching performance.

Feature-based training (Towler et al., 2021b) represents a significant departure from the philosophy of previous attempts to improve face identification through training, which have typically focused on the holistic processes involved in familiar face learning and recognition—albeit, to limited success (see Towler et al., 2021a for review). The successful application of this approach to matching masked faces adds to an emerging literature that feature-based training is a promising route to improving face matching performance generally (Towler et al., 2021a). These findings also support our initial proposition that interventions aimed at encouraging observers to extract maximal identity information from the available visual information, instead of those that seek to restore “normal” whole face processing, are uniquely suited to the challenge of improving the accuracy of masked face identification. Future research may explore whether other interventions based on this philosophy can also improve masked face identification. Further, the success of diagnostic feature training for masked faces—where holistic processing is disrupted (Freud et al., 2020; Stajduhar et al., 2021)—raises the possibility that a similar feature-based training might one day be beneficial for prosopagnosia patients whose face recognition deficits have been attributed to impairments in holistic processing (Avidan et al., 2011; Busigny et al., 2010; Levine & Calvanio, 1989; Ramon et al., 2010,2010,2010).

### Limitations

Although diagnostic feature training improved d′ and overall accuracy, the increase in AUC did not produce a significant interaction in the ANOVA. Notably, our sample size was determined by a power analysis with an expected interaction effect size of $\eta_{p}^{2}$ = 0.10, based on Towler et al. (2021b) reported effect size for unmasked faces ($\eta_{p}^{2}$ = 0.15). However, the ANOVA returned an interaction effect size of just $\eta_{p}^{2}$ = 0.04. Thus, despite following the hypothesised pattern, the interaction likely failed to reach significance due to our reduced statistical power to detect this smaller than expected effect. The discrepancy between the significant interaction for d′ and non-significant interaction for AUC, which are both measures of sensitivity, is likely due to the way they are calculated. AUC reflects the shape of the ROC that is plotted using hits and false alarms across varying response thresholds (i.e. our 6AFC scale), whereas d′ is calculated from a single threshold across all trials (Macmillan & Creelman, 2004). Future research with larger samples will reveal whether diagnostic feature training also improves AUC.

## Conclusion

The wearing of face masks in public poses significant challenges to face recognition (Freud et al., 2020), emotion recognition (Noyes et al., 2021), and face matching (Carragher & Hancock, 2020). Moreover, exposure to individuals wearing face masks over the course of the pandemic does not appear to have improved our ability to recognise masked faces (Freud et al., 2021). Yet, face masks are likely to remain a common sight in public spaces for the remainder of the COVID-19 pandemic, and perhaps beyond (Horii, 2014; Office for National Statistics, 2021). The current study shows that some of the deficit in masked face matching performance can be alleviated by training observers to compare the ears and any facial markings on the faces (Towler et al., 2021b). Even though face masks disrupt the holistic processing thought to underpin face recognition (Freud et al., 2020), diagnostic feature training offers an alternative route to improved face matching performance by engaging the featural processing strategies (Towler et al., 2017) that are associated with the superior abilities of professional facial examiners (Towler et al., 2021a; White et al., 2015, 2021,2021). This simple strategy could assist professional staff who are tasked with identifying masked faces in applied settings.

## Supplementary Information

**Additional file 1:** A complete report of our unsuccessful attempt to improve matching performance for the unmasked EFCT using the “feature-instruction” approach devised by Megreya and Bindemann (2018).

## Data Availability

The datasets analysed in the current study are available in the OSF repository [https://osf.io/9y24q/], as are those for the supplementary materials [https://osf.io/hszxr/].

## Abbreviations

EFCT: Expertise in facial comparison test
6AFC: 6-Alternative Forced Choice scale
ROC: Receiver operating characteristic
AUC: Area under the curve
OSF: Open science framework
ANOVA: Analysis of variance
RT: Response time

## Footnotes

1 We use “face mask” to mean any accessory or garment that covers the lower half of the face (e.g. nose, mouth, chin). A surgical/medical mask is a common type of “face mask”.

2 To account for removing one between-participant level in the current study (Towler et al., 2021b).

3 Regardless of final completion status, all data were excluded from participants who accessed the experiment more than once and started the face matching task on multiple occasions.

4 No participants were excluded for our other pre-registered exclusion criteria; taking longer than 60 min to complete the task or having a pre-training AUC of ≤ 0.48. This final exclusion criterion was set below 0.50 (chance responding) with the intention of only removing participants who did not follow or understand task instructions, rather than those who were not very good at the task.

## Declarations

### Open practices statement

Prior to data collection, we pre-registered the aims, hypotheses, design, and analyses for the current study [https://osf.io/qw27y] and supplementary materials [https://osf.io/y3ud2] on the OSF. The datasets generated and analysed in the current study [https://osf.io/9y24q/] and supplementary materials [https://osf.io/hszxr/] are also available in the OSF repository.
