# Supplementary material for: Masked face identification is improved by diagnostic feature training
Source: Cogn Res Princ Implic. 2022 Apr 5;7:30. doi: 10.1186/s41235-022-00381-x (PMC8980792; doi:10.1186/s41235-022-00381-x)
Supplement: Supplementary file 1 — Additional file 1: A complete report of our unsuccessful attempt to improve matching performance for the unmasked EFCT using the “feature-instruction” approach devised by Megreya and Bindemann (2018). [file 41235_2022_381_MOESM1_ESM.docx]

**Masked face identification is improved by diagnostic feature training**

*Daniel J. Carragher^1^, Alice Towler^2^, Viktoria R. Mileva^1^, David White^2^ & Peter J. B. Hancock^1^

****SUPPLEMENATRY MATERIALS****

^1^Psychology

Faculty of Natural Sciences

University of Stirling

Stirling, Scotland

United Kingdom

^2^School of Psychology

University of New South Wales

Sydney, New South Wales

Australia

Total Word Count: 2450 approx.

*Corresponding Author:

Daniel J. Carragher

School of Psychology

Faculty of Health and Medical Sciences

University of Adelaide

Adelaide, South Australia, 5000

[danieljcarragher@gmail.com](mailto:danieljcarragher@gmail.com)

**Background**

In our initial attempt to improve masked face matching performance, we conducted an experiment based on the feature instruction procedure first reported by Megreya and Bindemann (2018). In their study, Megreya and Bindemann (2018) investigated whether instructing observers to attend to either the eyes, eyebrows or ears of the faces they were comparing could improve face matching performance. This approach differs from the diagnostic feature training of Towler, Keshwa, Ton, Kemp, and White (2021) because observers were only given a very simple instruction to attend to a specific facial feature (e.g., “….*please focus on the eyes.*”), instead of receiving more detailed training. Across two experiments, Megreya and Bindemann (2018) reported that face matching performance improved when observers were instructed to attend to the eyebrows of the faces, remained constant when attending to the eyes, and decreased when attending to the ears.

In this pre-registered project, our overarching goal was to test whether this feature instruction approach could be used to improve masked face matching performance. We began, however, with a replication experiment, the aim of which was to see whether we could reproduce Megreya and Bindemann’s (2018) results using the EFCT (White, Phillips, Hahn, Hill, & O'Toole, 2015) and in an online setting. All faces were presented unmasked. We also added a control condition to the design. Participants in the control condition completed the face matching task without receiving the additional instructions to focus on a specific facial feature. To foreshadow our findings, we did not replicate the original pattern of results. For transparency, we report the results of this first experiment below.

**Method**

**Sample Size**

In their Experiment 1, Megreya and Bindemann (2018) reported a significant interaction for their measure of sensitivity (*d*′) between Instruction (pre-, post-) and Feature (eyes, eyebrows, ears) for own-race faces (Arab), with an effect size of $\text{η}_{\text{p}}^{\text{2}}$ = 0.23. Our *a priori* power analysis (G*Power; Faul, Erdfelder, Lang, & Buchner, 2007) indicated that a total sample of 108 participants would be required to achieve 95% power to detect an effect of $\text{η}_{\text{p}}^{\text{2}}$ = 0.15 in a mixed-measures ANOVA with a 2 level within-participants factor (*Instruction*: Pre-, Post-) and a 4 level between-participants factor (*Feature Condition*: eyes, eyebrows, ears, control) at a conventional alpha of α = .05. Thus, we required 27 participants per feature condition. This sample size is slightly higher than the 20 participants Megreya and Bindemann (2018) had in each of their feature instruction conditions.

Despite the power analysis suggesting that 27 participants would be required for sufficient power, we made the methodological decision to oversample each condition. This decision served several purposes including to pre-empt participant exclusions, reduce any additional noise that might be associated with online data collection, and to account for the possibility that replicated effect sizes might be smaller than those in the published literature (Open Science Collaboration, 2015). Therefore, we aimed to recruit 40 participants to each feature condition (eyes, eyebrows, ears, control), so that we would have data from between 30-35 participants in each condition for the final analysis.

**Participants**

We recruited 160 participants who completed the experiment from the online research platform *Prolific*. All participants were aged 18 years or older and reported living in the UK. To maintain data integrity, we applied several pre-registered exclusion criteria to the collected data prior to analysis. Participants who attempted to complete the experiment more than once (*n* = 9)^[[1]](#footnote-1)^, and those that took longer than 60 minutes (*n* = 5) to complete the task were excluded. Participants who failed the attention check question (*n* = 2) or attention check trials (*n* = 3) were also excluded from all analyses^[[2]](#footnote-2)^.

The final sample consisted of 141 participants: 34 participants in the eye feature condition (22 female, 12 male, *M*_age_ = 31.8, *SD* = 10.2, range = 19-57), 35 participants in the eyebrow feature condition (24 female, 11 male, *M*_age_ = 35.7, *SD* = 13.9, range = 19-65), 37 participants in the ear feature condition (21 female, 16 male, *M*_age_ = 36.5, *SD* = 12.4, range 18-60), and 35 participants in the control condition (20 female, 14 male, 1 other, *M*_age_ = 36.9, *SD* = 14.9, range 18-68). This research was approved by the General University Ethics Panel at the University of Stirling. All participants gave their informed consent before starting the experiment, were debriefed on completion, and were reimbursed £2.50 for their time.

**Materials**

***Expertise in Facial Comparison Test***

Participants completed the EFCT (White et al., 2015) as described in the main text. However, all faces were presented unmasked in the current experiment.

***Attention Checks***

This experiment included two attention checks. The first was the attention check (famous face pairs) described in the main text. The second occurred immediately after participants received their specific feature condition instructions midway through the experiment. The next screen asked the participants to identify the facial feature that they had just been instructed to focus on. The question was multiple choice, with the correct answer shown along with two incorrect alternatives (“forehead” and “hair” - neither of which were feature conditions). Although incorrect responses were immediately corrected with feedback (“*Incorrect! Please focus on the [feature condition] of the two faces*”), all data from participants that gave an incorrect response to this attention check were discarded out of an abundance of caution. Participants in the control condition were asked to identify what type of images were shown in the current experiment (“Faces”, “Cars” or “Bikes”), and those who failed to respond “Faces” had their data discarded from all analyses.

**Procedure**

The experimental procedure was very similar to that reported in the main text, with the exception of the feature instruction manipulation. Participants were randomly allocated into one of four feature instruction conditions (eyes, eyebrows, ears, control). After completing the first half of the EFCT, participants were presented with a new set of instructions that emphasised that they should use a particular facial feature to assist with their matching decisions. These instructions were closely based on those used by Megreya and Bindemann (2018). The instructions followed the same format in all feature conditions (eyes, eyebrows, ears): “*Now you are going to continue the task. This time, please focus on the [feature condition] of the two faces (e.g., characteristic 1, characteristic 2). Compare the appearance of the [feature condition] between the images before making your final identity decision*”. The example characteristics given in the second sentence were designed to prompt the participants to think about how they might compare the feature between faces. These example characteristics were based on the attributes that Abudarham and Yovel (2016) reported to be perceptually salient about each feature: the colour and the shape of the eyes, the thickness and the shape of the eyebrows, and the size and the shape of the ears. Participants in the control condition were simply given a repeat of the task instructions that all participants received at the start of the experiment: “*Now you are going to continue the task. Please compare the appearance of the two faces before making your final identity decision”*. All participants then completed the second half of the EFCT. The experiment took an average of 19 minutes (*SD* = 8.6) to complete.

**Analysis**

This experiment was also pre-registered on the OSF prior to data collection [https://osf.io/y3ud2]. Analyses were performed as described in the main text. All ANOVAs reported below have Instruction (pre-, post-) as a within-participants factor and Feature Condition (eyes, eyebrows, ears, control) as a between-participants factor. All data analysed in this study are available on the OSF [https://osf.io/hszxr/].

**Results**

**Primary Analyses**

***AUC***

A mixed measures ANOVA on AUC showed that the main effects of Instruction, *F*(1, 137) = 2.91, *p* = .090, $\text{η}_{\text{p}}^{\text{2}}$ = .02, and Feature Condition were non-significant, *F*(3, 137) = 2.40, *p* = .071, $\text{η}_{\text{p}}^{\text{2}}$ = .05 (see Figure S1). Crucially, the interaction between the two factors was significant, *F*(3, 137) = 2.78, *p* = .044, $\text{η}_{\text{p}}^{\text{2}}$ = .06. However, an analysis of simple main effects revealed that the only significant change in AUC was a post-instruction *decrease* for participants in the eye condition (see Table S1). Equivalent Bayesian analyses provide strong support (Lee & Wagenmakers, 2014) for the notion that attending to the eyes impairs face matching performance. Data in the remaining conditions provided anecdotal (control) or moderate evidence (eyebrows, ears) in favour of the null hypothesis (i.e., that these instructions do not affect face matching performance).


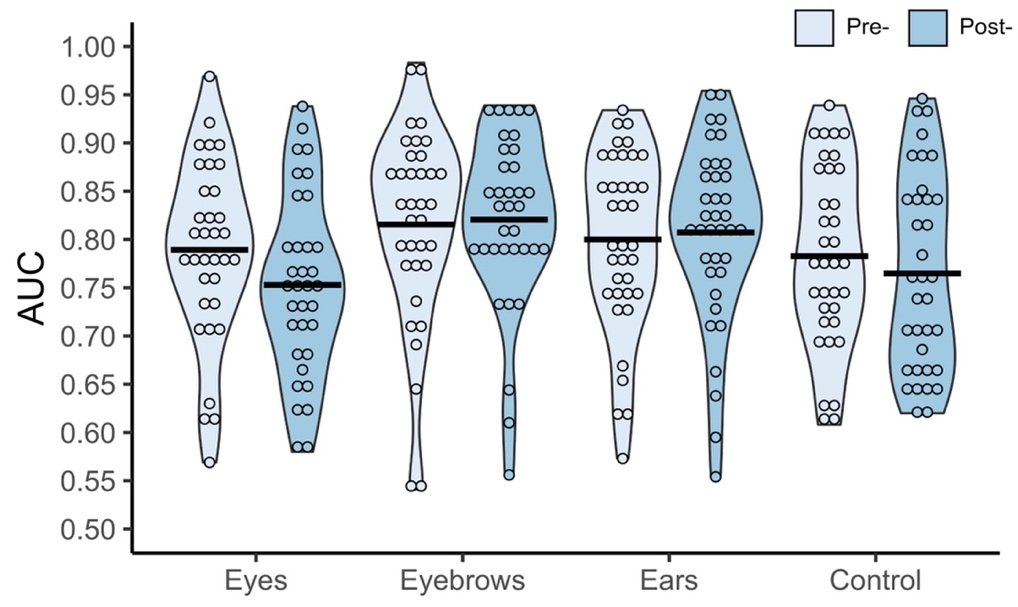


**Figure S1.** AUC pre- and post-instruction in each feature instruction condition. Unfilled circles represent individual data points (visualised in 1/30 bins by default), while the horizontal black lines represent the mean.

**Table S1**

Simple main effects comparing AUC pre-instruction and post-instruction for each feature condition. The Bonferroni corrected alpha level for four comparisons is *p* = .0125.

| **Feature** | **Pre-instruction**  **Mean (SD)** | **Post-instruction**  **Mean (SD)** | ***df*** | ***t*** | **95%CI** | ***p*** | ***d*** | **BF_10_** |
| --- | --- | --- | --- | --- | --- | --- | --- | --- |
| Eyes | .789 (.093) | .753 (.093) | 33 | 3.12 | .013, .060 | .004* | 0.54 | 10.09 |
| Eyebrows | .815 (.102) | .821 (.090) | 34 | -0.37 | -.033, .023 | .714 | -0.06 | 0.19 |
| Ears | .800 (.093) | .807 (.093) | 36 | -0.55 | -.034, .019 | .586 | -0.09 | 0.20 |
| Control | .783 (.093) | .765 (.102) | 34 | 1.69 | -.004, .040 | .100 | 0.29 | 0.66 |

*Identifies statistically significant comparisons.

***Criterion***

A mixed measures ANOVA revealed a significant main effect of Instruction, *F*(1, 137) = 64.11, *p* < .001, $\text{η}_{\text{p}}^{\text{2}}$ = .32 (see Figure S2), with all feature conditions showing a larger response bias pre-instruction (*M* = 0.23, *SD* = 0.44) than post-instruction (*M* = -0.01, *SD* = 0.44). Neither the main effect of Feature Condition, *F*(3, 137) = 1.31, *p* = .274, $\text{η}_{\text{p}}^{\text{2}}$ = .03, or the interaction were significant, *F*(3, 137) = 1.97, *p* = .121, $\text{η}_{\text{p}}^{\text{2}}$ = .04.


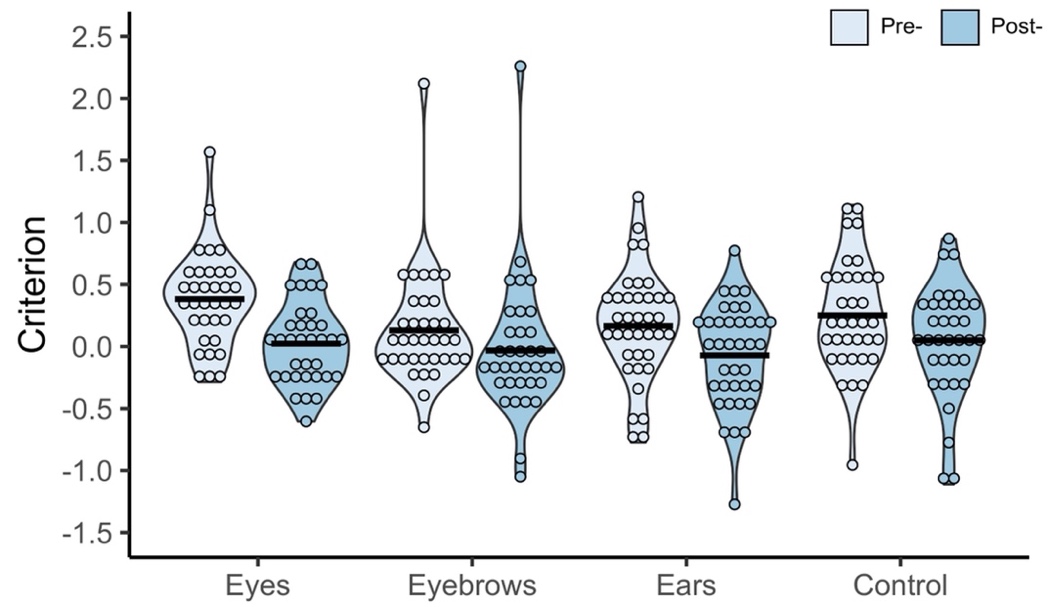


**Figure S2.** Response bias pre- and post-instruction for each feature condition. Positive values correspond to a conservative response bias (“mismatch”), while negative values represent a liberal bias (“match”).

A series of one-sample *t*-tests revealed that pre-instruction response bias differed significantly from neutral responding for the eyes, ears and control conditions. Response bias did not differ from neutral for any feature condition post-instruction (see Table S2).

**Table S2**

A series of one sample *t*-tests comparing the response bias shown by each feature condition to 0, in order to determine whether it differed from neutral responding.

|  | **Pre-Instruction** | | | | | | **Post-Instruction** | | | | | |
| --- | --- | --- | --- | --- | --- | --- | --- | --- | --- | --- | --- | --- |
|  | ***M(SD)*** | ***df*** | ***t*** | **95%CI** | ***p*** | ***d*** | **M(SD)** | ***df*** | ***t*** | **95%CI** | ***p*** | ***d*** |
| Eyes | 0.38 (0.38) | 33 | 5.85 | 0.25, 0.52 | <.001* | 1.00 | 0.02 (0.32) | 33 | 0.42 | -0.09, 0.14 | .679 | 0.07 |
| Eyebrows | 0.13 (0.46) | 34 | 1.67 | -0.03, 0.29 | .105 | 0.28 | -0.03 (0.55) | 34 | -0.35 | -0.22, 0.16 | .730 | -0.06 |
| Ears | 0.17 (0.44) | 36 | 2.32 | 0.02, 0.31 | .026* | 0.38 | -0.07 (0.41) | 36 | -1.06 | -0.21, 0.07 | .298 | -0.17 |
| Control | 0.25 (0.45) | 34 | 3.30 | 0.10, 0.41 | .002* | 0.56 | 0.05 (0.45) | 34 | 0.65 | -0.11, 0.20 | .519 | 0.11 |

*Identifies statistically significant comparisons.

**Secondary Analyses**

***Sensitivity***

A mixed measures ANOVA on d′ revealed no significant main effects: Instruction, *F*(1, 137) = 0.85, *p* = .357, $\text{η}_{\text{p}}^{\text{2}}$ = .01, Feature Condition, *F*(3, 137) = 1.69, *p* = .172, $\text{η}_{\text{p}}^{\text{2}}$ = .04. The interaction was also non-significant, *F*(3, 137) = 2.61, *p* = .054, $\text{η}_{\text{p}}^{\text{2}}$ = .05 (see Figure S3).


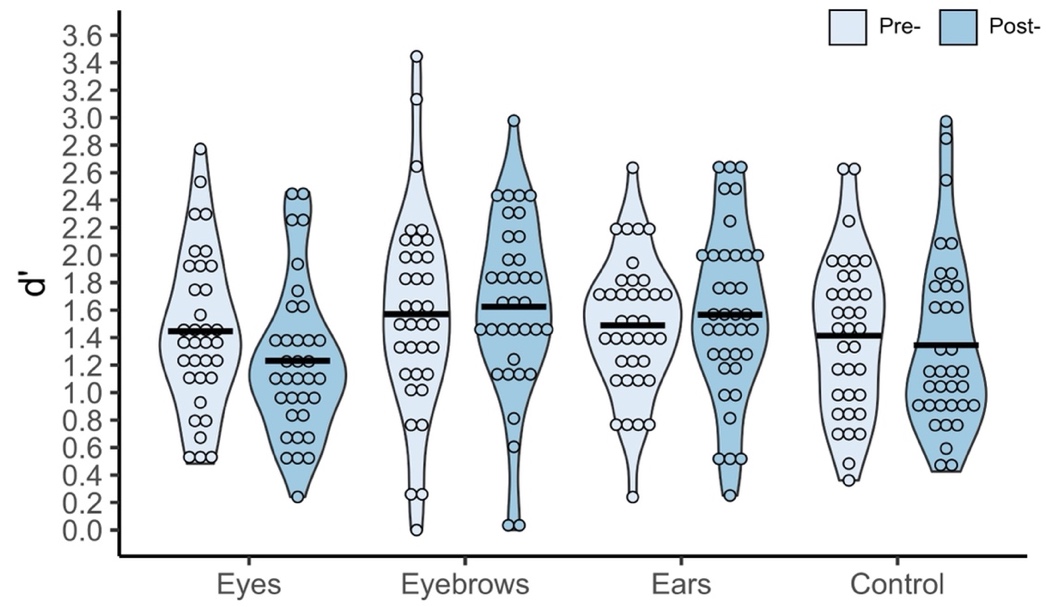


**Figure S3.** Sensitivity (d′) pre- and post-instruction for each feature condition.

***Accuracy***

**Overall.** Neither the main effects of Instruction, *F*(1, 137) = 0.01, *p* = .921, $\text{η}_{\text{p}}^{\text{2}}$ = .00, or Feature Condition were significant, *F*(3, 137) = 2.04, *p* = .111, $\text{η}_{\text{p}}^{\text{2}}$ = .04. Their interaction was also non-significant, *F*(3, 137) = 1.09, *p* = .357, $\text{η}_{\text{p}}^{\text{2}}$ = .02 (see Figure S4).


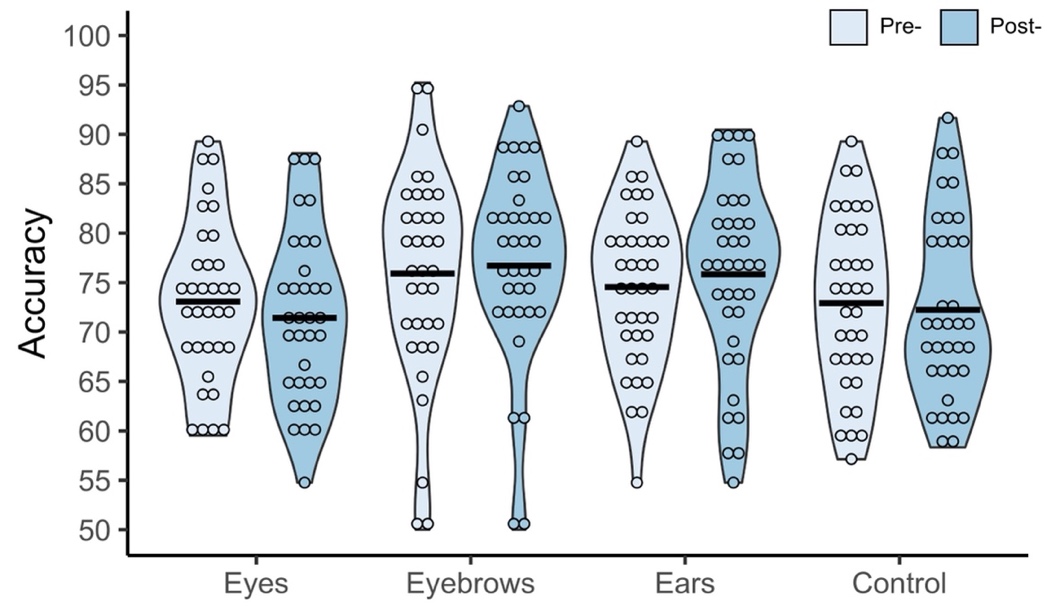


**Figure S4.** Overall accuracy (%) pre- and post-instruction in each feature condition.

**Match Trials.** The main effect of Instruction was significant, *F*(1, 137) = 33.70, *p* < .001, $\text{η}_{\text{p}}^{\text{2}}$ = .20, with higher accuracy post-instruction (*M* = 74.4%, *SD* = 15.9) than pre-instruction (*M* = 67.6%, *SD* = 16.8). The main effect of Feature Condition was also significant, *F*(3, 137) = 3.09, *p* = .029, $\text{η}_{\text{p}}^{\text{2}}$ = .06, as a result of higher average accuracy for the eyebrow (*M* = 75.3%) and ear (*M* = 74.1%) conditions, compared to the eye (*M* = 66.7%) and control (*M* = 67.9%) conditions. The interaction between Instruction and Feature Condition was non-significant, *F*(3, 137) = 0.69, *p* = .561, $\text{η}_{\text{p}}^{\text{2}}$ = .02 (see Figure S5).


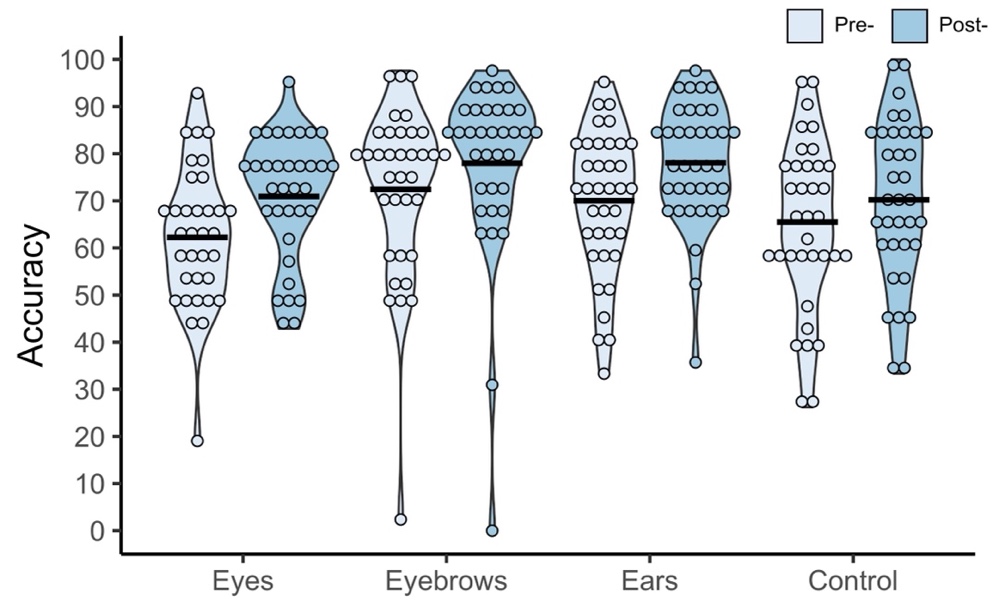


**Figure S5.** Match trial accuracy (%) pre- and post-instruction for each feature condition.

**Mismatch Trials.** The main effect of Instruction was significant, *F*(1, 137) = 47.42, *p* < .001, $\text{η}_{\text{p}}^{\text{2}}$ = .26, with accuracy higher pre-instruction (*M* = 80.6%, *SD* = 13.0) than post-instruction (*M* = 73.8%, *SD* = 15.3). The main effect of Feature Condition not significant, *F*(3, 137) = 0.09, *p* = .966, $\text{η}_{\text{p}}^{\text{2}}$ = .00. The interaction between the two factors was significant, *F*(3, 137) = 3.05, *p* = .031, $\text{η}_{\text{p}}^{\text{2}}$ = .06 (see Figure S6). An analysis of simple main effects revealed that accuracy declined significantly after receiving feature instructions for participants in the eye (*F* = 35.9, *p* < .001), ear (*F* = 7.0, *p* = .012) and control conditions (*F* = 13.8, *p* < .001), but not the eyebrow condition (*F* = 3.2, *p* = .084).


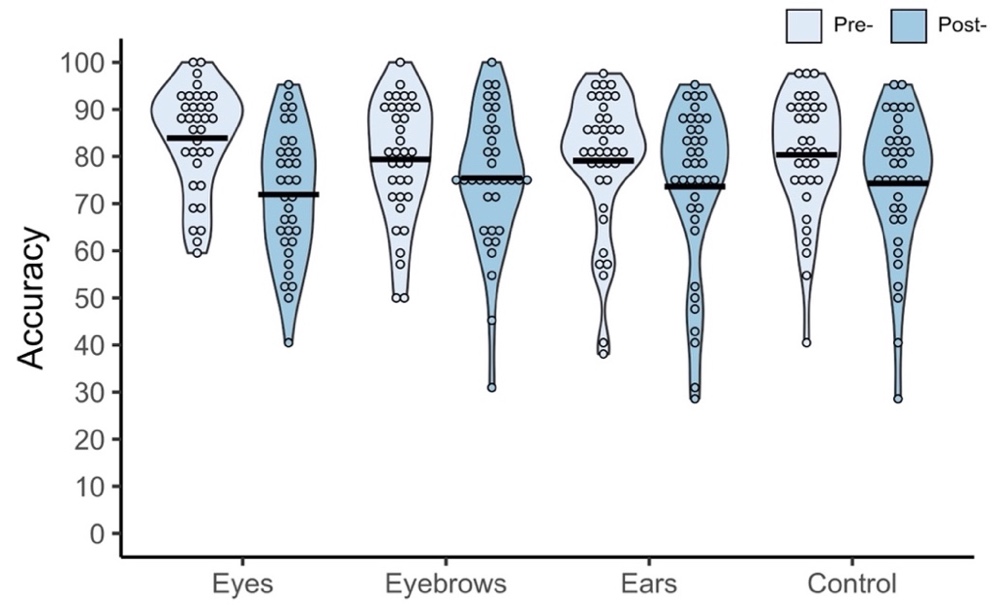


**Figure S6.** Mismatch trial accuracy (%) pre- and post-instruction for each feature condition.

***Response Time***

Finally, we investigated whether any differences in response behaviour could be attributed to a change in median response time (RT) post-instruction. The main effect of Instruction was significant, *F*(1, 137) = 25.68, *p* < .001, $\text{η}_{\text{p}}^{\text{2}}$ = .16, which showed that participants were faster to respond post-instructions (*M* = 3.46 secs, *SD* = 1.61) than pre-instruction (*M* = 3.89 secs, *SD* = 1.84). The main effect of Feature Condition was non-significant, *F*(3, 137) = 1.66, *p* = .178, $\text{η}_{\text{p}}^{\text{2}}$ = .04. The interaction between the two factors was significant, *F*(3, 137) = 4.04, *p* = .009, $\text{η}_{\text{p}}^{\text{2}}$ = .08. An analysis of simple main effects revealed that RT decreased post-instruction for participants in the eye (*F* = 13.01, *p* = .001), eyebrow (*F* = 8.51, *p* = .006) and control (*F* = 36.26, *p* < .001) conditions, but not for those in the ear instruction condition (*F* = 0.02, *p* = .901).

**Discussion**

These data do not replicate the pattern of results reported by Megreya and Bindemann (2018). The only significant change in sensitivity (AUC) occurred for participants who were instructed to attend to the eyes, but their performance fell significantly. This non-replication occurred despite closely following the procedure reported in the original study and recruiting a larger sample in each condition. Overall, these data provide no support for the efficacy of basic feature instructions to improve face matching accuracy, potentially raising questions about the generalisability of this effect beyond the original study (Megreya & Bindemann, 2018).

However, it is important to acknowledge that this experiment differs from the original in several ways that could potentially have contributed to our non-significant results. First, our stimulus set differs from that used in the original study. Instructions to attend to particular facial features might be uniquely affected by the presence or prominence of those features in any given stimulus set. Moreover, the original study used exclusively male face stimuli with the background removed (by cropping around the outline of the head); our stimulus set contained both male and female faces, with the background in place. Finally, we also tested participants in an online setting, whereas Megreya and Bindemann’s (2018) participants were lab-based. Any one of these differences might explain why we failed to replicate the original pattern of results. Future research is needed to clarify under which conditions feature instructions lead to improved face matching performance.

The original aim of our project was to investigate whether feature instructions could improve masked face matching performance. But a non-replication of the original effect with unmasked faces was noted in our pre-registration as rationale for potentially altering our approach to this project. As a consequence of these data, we shifted focus to test whether Towler, Keshwa, et al.’s (2021) diagnostic feature training would improve masked face matching performance. Crucially, Towler, Keshwa, et al. (2021) had already shown that their diagnostic feature training course improves face matching performance on the EFCT with unmasked faces (Experiment 1), and in an online experiment (Experiment 2). As such, all participants in the main text completed a matching task with masked faces, because any deviation from Towler, Keshwa, et al.’s (2021) results could already be attributed to the masks themselves.

**References**

Abudarham, N., & Yovel, G. (2016). Reverse engineering the face space: Discovering the critical features for face identification. *J Vis, 16*(3), 40-40. doi:10.1167/16.3.40

Faul, F., Erdfelder, E., Lang, A.-G., & Buchner, A. (2007). G* Power 3: A flexible statistical power analysis program for the social, behavioral, and biomedical sciences. *Behavior Research Methods, 39*(2), 175-191. doi:10.3758/bf03193146

Lee, M. D., & Wagenmakers, E.-J. (2014). *Bayesian cognitive modeling: A practical course*: Cambridge University Press.

Megreya, A. M., & Bindemann, M. (2018). Feature instructions improve face-matching accuracy. *PLoS One, 13*(3), e0193455. doi:10.1371/journal.pone.0193455

Open Science Collaboration. (2015). Estimating the reproducibility of psychological science. *Science, 349*(6251), aac4716. doi:10.1126/science.aac4716

Towler, A., Keshwa, M., Ton, B., Kemp, R., & White, D. (2021). Diagnostic feature training improves face matching accuracy. *Journal of Experimental Psychology: Learning, Memory, and Cognition*. doi:10.1037/xlm0000972

White, D., Phillips, P. J., Hahn, C. A., Hill, M., & O'Toole, A. J. (2015). Perceptual expertise in forensic facial image comparison. *Proceedings of the Royal Society B: Biological Sciences, 282*(1814), 20151292. doi:10.1098/rspb.2015.1292

1. A technical issue allowed some participants to access the experiment more than once. As per the main text, we excluded all data from participants who accessed the experiment more than once and started the face matching task on more than one occasion. [↑](#footnote-ref-1)
2. No participants were excluded for our other pre-registered exclusion criteria: taking less than 6 minutes to complete the task, or for having an AUC of ≤ 0.45. [↑](#footnote-ref-2)
